# Stability Analysis of an Improved HBV Model with CTL Immune Response

**DOI:** 10.1155/2014/407272

**Published:** 2014-10-29

**Authors:** Mostafa Khabouze, Khalid Hattaf, Noura Yousfi

**Affiliations:** ^1^Department of Mathematics and Computer Science, Faculty of Sciences Ben M'sik, Hassan II University, P.O. Box 7955, Sidi Othman, Casablanca, Morocco; ^2^Centre Régional des Métiers de l'Education et de la Formation (CRMEF), Derb Ghalef, 20340 Casablanca, Morocco

## Abstract

To better understand the dynamics of the hepatitis B virus (HBV) infection, we introduce an improved HBV model with standard incidence function, cytotoxic T lymphocytes (CTL) immune response, and take into account the effect of the export of precursor CTL cells from the thymus and the role of cytolytic and noncytolytic mechanisms. The local stability of the disease-free equilibrium and the chronic infection equilibrium is obtained via characteristic equations. Furthermore, the global stability of both equilibria is established by using two techniques, the direct Lyapunov method for the disease-free equilibrium and the geometrical approach for the chronic infection equilibrium.

## 1. Introduction

Currently, HBV infection is a major global health problem, which can lead to cirrhosis and liver cancer. From the World Health Organization (WHO), more than 240 million people have chronic (long-term) liver infections, and about 600,000 people die every year due to the acute or chronic consequences of hepatitis B [1]. Therefore, many mathematical models have been developed in order to understand the dynamics of HBV infection. In this paper, we consider the model presented by Pang et al. in [2] that is given by the following nonlinear system of differential equations:

(1) x˙=λ−dx−βvx+qyz,y˙=βvx−ay−(p+q)yz,v˙=ky−uv,z˙=b+cyzω+y−μz,

where *x*(*t*), *y*(*t*), *v*(*t*), and *z*(*t*) are the numbers of uninfected target cells, infected cells, free virus, and CTL cells at time *t*, respectively. Susceptible host (healthy hepatocytes) cells are produced at a rate *λ*, die at a rate *dx*, and become infected by virus at a rate *βxv*. Infected cells die at a rate *ay*, return to the uninfected state by a nonlytic effector mechanism [3] at a rate *qyz*, and are killed by the CTL immune response at a rate *pyz*. Free virus is produced by infected cells at a rate *ky* and decays at a rate *uv*. CTL cells expand in response to viral antigen derived from infected cells at a rate *cyz*/(*ω* + *y*), where *c* is HBV-specific CTL stimulation rate and *ω* represents virus load for half-maximal CTL cells stimulation [4] and decay in the absence of antigenic stimulation at a rate *μz*. The parameter *b* represents the export of precursor CTL cells from the thymus [4]. Note that the CTL immune response plays an important role in antiviral defense by killing infected cells and its effect has recently drawn much attention of many authors (see, e.g., [5–10]).

On the other hand, the authors Pang et al. [2] determined the basic reproduction number of system (1) as follows:

(2) $$\begin{array}{c} R_{0}^{*}=\frac{\lambda }{d}\frac{\beta k}{u\left(a+\left(p+q\right)\left(b/\mu \right)\right)}. \end{array}$$

As in [10, 11], we observe that *R*_0_^*^ is proportional to *λ*/*d* which represents the number of total cells of the liver. This suggests that (1) may not be a reasonable model for describing HBV virus infection since it implies that an individual with a smaller liver may be more resistant to the virus infection than an individual with a larger one. Therefore, we propose the following model:

(3) x˙=λ−dx−βvxx+y+qyz,y˙=βvxx+y−ay−(p+q)yz,v˙=ky−uv,z˙=b+cyzω+y−μz.

In our case, the basic reproduction number is

(4) $$\begin{array}{c} R_{0}=\frac{\beta k}{u\left(a+\left(p+q\right)\left(b/\mu \right)\right)}, \end{array}$$

which is independent of liver size.

The rest of this paper is organized as follows. In the next section, we show that our model is well posed by proving the existence, positivity, and boundedness of solutions of problem. Further, we determine the steady states of the model. In Section 3, we discuss the local stability of equilibria by analyzing the corresponding characteristic equations. The global stability of equilibria is analyzed in Section 4. The paper ends with a conclusion and discussion in Section 5.

## 2. Well Posedness and Steady States

In this section, we will establish the positivity and boundedness of solutions of model (3), which imply that our model is well posed. Further, we will determine the steady states of the model.

### 2.1. Positivity and Boundedness of Solutions

First, we have the following result.

**Theorem 1 .**

All solutions starting from nonnegative initial conditions exist for all *t* > 0 and remain bounded and nonnegative. Moreover, we have

(i) *T*(*t*) ≤ *T*(0) + *λ*/*δ*,
(ii) *v*(*t*) ≤ *v*_0_ + (*k*/*u*)||*y*||_*∞*_,
(iii) *z*(*t*) ≤ *z*_0_ + *b*/*μ* + (*c*/*pω*)(*λ*/*μ* + *x*_0_ + *y*_0_ + max⁡(0,1 − *d*/*μ*)||*x*||_*∞*_ + max⁡(0,1 − *a*/*μ*)||*y*||_*∞*_),

where *T* = *x* + *y* that represents the total cells of liver and *δ* = min⁡(*a*, *d*).

**Proof**

For the positivity, we show that any solution starting in nonnegative orthant *ℝ*_+_^4^ = {(*x*, *y*, *v*, *z*) ∈ *ℝ* ^4^ : *x* ≥ 0, *y* ≥ 0, *v* ≥ 0, *z* ≥ 0} remains there forever. In fact, (*x*(*t*), *y*(*t*), *v*(*t*), *z*(*t*)) ∈ *ℝ*_+_^4^ and we have

(5) x˙x=0=λ+qyz≥0,y˙y=0=βv≥0,v˙v=0=ky≥0,z˙z=0=b>0.

Hence, the positivity of all solutions initiating in *ℝ*_+_^4^ is guaranteed.

Now, we prove that the solutions are bounded.

As $\dot{T}\leq \lambda -dx-ay$, we deduce that

(6) $$\begin{array}{c} T\left(t\right)\leq T\left(0\right)e^{-\delta t}+\frac{\lambda }{\delta }\left(1-e^{-\delta t}\right), \end{array}$$

since 0 ≤ *e* ^−*δt*^ ≤ 1 and 1 − *e* ^−*δt*^ ≤ 1, we get (i).

Next, we show (ii). The equation, $\dot{v}=ky-uv$, implies that

(7) $$\begin{array}{c} v\left(t\right)=v_{0}e^{-ut}+k\overset{t}{\underset{0}{\int }}y\left(s\right)e^{(s-t)u}ds. \end{array}$$

Then,

(8) $$\begin{array}{c} v\left(t\right)\leq v_{0}+\frac{k}{u}{||y||}_{\infty }\left(1-e^{-tu}\right). \end{array}$$

Since 1 − *e* ^−*tμ*^ ≤ 1, we deduce (ii).

Finally, we show (iii). From the fourth equation of (3), we get

(9) $$\begin{array}{c} \dot{z}+\mu z\leq b+\frac{c}{\omega }yz. \end{array}$$

Hence,

(10) $$\begin{array}{c} \dot{z}+bz\leq b+\frac{c}{p\omega }\left[\lambda -\left(\dot{x}+dx\right)-\left(\dot{y}+ay\right)\right]. \end{array}$$

Thus,

(11) zteμt−z0≤bμ+cλpωμeμt−1−cpω∫0teμ−ds∫0teμ−dsddsxsedsds+∫0teμ−asddsyseasds.

Using the integration by parts, we get

(12) ∫0teμ−dsddsxsedsds  =xseμs0t−μ−d∫0txseμsds,∫0teμ−asddsyseasds  =xseμs0t−μ−a∫0tyseμsds.

Hence,

(13) zt≤cpωx0+y0+z0e−μt+bμ+cλpωμ1−e−μt +cpω∫0tμ−dxs+μ−ayseμs−tds    −xt−yt∫0t.

If *μ* − *d* ≤ 0 and *μ* − *a* ≤ 0, we have

(14) $$\begin{array}{c} z\left(t\right)\leq z_{0}+\frac{b}{\mu }+\frac{c}{p\omega }\left(\frac{\lambda }{\mu }+x_{0}+y_{0}\right). \end{array}$$

If *μ* − *d* ≤ 0 and *μ* − *a* ≥ 0, we have

(15) $$\begin{array}{c} z\left(t\right)\leq z_{0}+\frac{b}{\mu }+\frac{c}{p\omega }\left[\frac{\lambda }{\mu }+x_{0}+y_{0}+\left(1-\frac{a}{\mu }\right){||y||}_{\infty }\right]. \end{array}$$

If *μ* − *d* ≥ 0 and *μ* − *a* ≤ 0, we have

(16) $$\begin{array}{c} z\left(t\right)\leq z_{0}+\frac{b}{\mu }+\frac{c}{p\omega }\left[\frac{\lambda }{\mu }+x_{0}+y_{0}+\left(1-\frac{d}{\mu }\right){||x||}_{\infty }\right]. \end{array}$$

If *μ* − *d* ≥ 0 and *μ* − *a* ≥ 0, we have

(17) zt≤z0+bμ+cpωλμ+x0+y0+1−dμx∞ +1−aμy∞.

From (14)–(17), we deduce (iii).

### 2.2. Steady States

In this subsection, we show that there exist a disease-free equilibrium and one infection equilibrium which represents the chronic infection equilibrium.

It is not hard to see that if *R*_0_ ≤ 1, the disease-free steady state *E*_*f*_(*λ*/*d*, 0,0, *b*/*μ*) is the unique steady state, corresponding to the extinction of the free virus. The following result presents the existence and uniqueness of endemic equilibrium when *R*_0_ > 1.

**Theorem 2 .**

(1) If *R*_0_ ≤ 1, then the system (3) has a unique disease-free equilibrium of the form *E*_*f*_(*λ*/*d*, 0,0, *b*/*μ*).
(2) If *R*_0_ > 1, then the system (3) has a unique chronic infection equilibrium of the form *E* ^*^(*x* ^*^, *y* ^*^, *v* ^*^, *z* ^*^) with *x* ^*^ > 0, *y* ^*^ > 0, *v* ^*^ > 0, and *z* ^*^ > 0.

**Proof**

At any equilibrium, the following equations hold:

(18) $$\begin{array}{c} \lambda -dx-\frac{\beta vx}{x+y}+qyz=0,\\ \frac{\beta vx}{x+y}-ay-\left(p+q\right)yz=0,\\ ky-uv=0,\\ b+\frac{cyz}{\omega +y}-\mu z=0. \end{array}$$

By (18), we get

(19) v=kyu,z=bω+yμω+μ−cy,x=fy,

where

(20) $$\begin{array}{c} f\left(y\right)=\frac{\lambda }{d}-y\left(\frac{a}{d}+\frac{pb\left(\omega +y\right)}{d\mu \omega +d\left(\mu -c\right)y}\right). \end{array}$$

Hence, we obtain the following equation:

(21) $$\begin{array}{c} \frac{\beta k}{u}\frac{f\left(y\right)}{f\left(y\right)+y}=a+\frac{b\left(p+q\right)\left(\omega +y\right)}{\mu \omega +\left(\mu -c\right)y}. \end{array}$$

Now, we consider the function *g* defined on [0, +*∞*[−{*μω*/(*c* − *μ*)} by

(22) $$\begin{array}{c} g\left(y\right)=\frac{\beta k}{u}\frac{f\left(y\right)}{f\left(y\right)+y}-a-\frac{b\left(p+q\right)\left(\omega +y\right)}{\mu \omega +\left(\mu -c\right)y}. \end{array}$$

We have *g*(0) = (*βk*/*u*)(1 − 1/*R*_0_) > 0 and

(23) f'y=−ad+pbω+ydμω+dμ−cy−dcpbωydμω+dμ−cy2<0,g'y=βkyf'y−fyufy+y2−p+qcbωyμω+μ−cy2<0.

Let *α* = *μω*/(*c* − *μ*) be a pole of *g*; then, we discuss two cases.

(i) If *c* > *μ*, then *α* > 0. As *z* = *b*(*ω* + *y*)/(*μω* + (*μ* − *c*)*y*) ≥ 0 we deduce that *y* < *α*. Hence there is no equilibrium point if *y* ≥ *α*. It is easy to show that
  (24) $$\begin{array}{c} \underset{y\to \alpha^{-}}{\lim}g\left(y\right)=-\infty . \end{array}$$
 Then the function *g* admits a unique root *y* ^*^ on interval ]0, *α*[, since *f*(0) = *λ*/*d* > 0 and lim⁡_*y*→*α*^−^_ *f*(*y*) = −*∞*. So, there exists a unique $\tilde{y}\in ]0,\alpha [$ such that $f(\tilde{y})=0$. We have
  (25) $$\begin{array}{c} g\left(\tilde{y}\right)=-a-\frac{b\left(p+q\right)\left(\omega +\tilde{y}\right)}{\mu \omega +\left(\mu -c\right)\tilde{y}}<0. \end{array}$$
 We deduce that $0<y^{*}<\tilde{y}$; this implies that $f(\tilde{y})<f(y^{*})<f(0)$ because *f* is decreasing on ]0, *α*[. Then 0 < *x* ^*^ < *λ*/*d*. Clearly *v* ^*^ and *z* ^*^ are positive.
 Hence, there exists a unique endemic *E* ^*^(*x* ^*^, *y* ^*^, *v* ^*^, *z* ^*^) with $y^{*}\in ]0,\tilde{y}[$, *x* ^*^ > 0, *v* ^*^ > 0, and *z* ^*^ > 0.
(ii) If *c* < *μ*, then *α* < 0 and lim⁡_*y*→+*∞*_ *f*(*y*) = −*∞*. Since *f*(0) = *λ*/*d* > 0, hence there exists a unique $\bar{y}\in ]0,+\infty [$ such that $f(\bar{y})=0$.
 We have
  (26) $$\begin{array}{c} g\left(\bar{y}\right)=-a-\frac{b\left(p+q\right)\left(\omega +\bar{y}\right)}{\mu \omega +\left(\mu -c\right)\bar{y}}<0. \end{array}$$
 Using the same technique, we deduce that *x* ^*^, *v* ^*^, and *z* ^*^ are positive.
 Thus, there exists a unique endemic *E* ^*^(*x* ^*^, *y* ^*^, *v* ^*^, *z* ^*^) with $y^{*}\in ]0,\bar{y}[$, *x* ^*^ > 0, *v* ^*^ > 0, and *z* ^*^ > 0.

This proves the theorem.

## 3. Local Stability of Equilibria

Let *E*(*x*, *y*, *v*, *z*) be any arbitrary equilibrium. Then the characteristic equation about *E* is given by

(27) $$\begin{array}{c} \left|\begin{array}{cccc} -d-\frac{\beta vy}{{\left(x+y\right)}^{2}}-\xi & \frac{\beta xv}{{\left(x+y\right)}^{2}}+qz & -\frac{\beta x}{x+y} & qy\\ \frac{\beta vy}{{\left(x+y\right)}^{2}} & -\frac{\beta xv}{{\left(x+y\right)}^{2}}-a-\left(p+q\right)z-\xi & \frac{\beta x}{x+y} & -\left(p+q\right)y\\ 0 & k & -u-\xi & 0\\ 0 & \frac{c\omega z}{{\left(\omega +y\right)}^{2}} & 0 & \frac{cy}{\omega +y}-\mu -\xi \end{array}\right|=0. \end{array}$$

The characterization of the local stability of the disease-free equilibrium is given by the following statement.

**Theorem 3 .**

Let us define *R*_0_ = *βk*/(*u*(*a* + (*p* + *q*)(*b*/*μ*))).

(i) If *R*_0_ < 1, then *E*_*f*_ is locally asymptotically stable.
(ii) If *R*_0_ > 1, then *E*_*f*_ is unstable.

**Proof**

At *E*_*f*_, (27) reduces to

(28) $$\begin{array}{c} \left(\mu +\xi \right)\left(\xi +d\right)\left[\xi^{2}+\left(u+\frac{\beta k}{uR_{0}}\right)\xi +\frac{\beta k}{R_{0}}\left(1-R_{0}\right)\right]=0, \end{array}$$

where the roots are

(29) ξ1=−μ,ξ2=−d,ξ3=u+βkuR02−4βkR01−R0−u+βkuR0 −u+βkuR02−4βkR01−R02−1,ξ4=u+βkuR02−4βkR01−R0−u+βkuR0 +u+βkuR02−4βkR01−R02−1.

It is clear that *ξ*_1_, *ξ*_2_, and *ξ*_3_ are negative. Moreover, *ξ*_4_ is negative when *R*_0_ < 1; thus, *E*_*f*_ is locally asymptotically stable.

Now, we focus on local stability of the chronic infection equilibrium *E* ^*^. It is easy to verify that the point *E* ^*^ does not exist if *R*_0_ < 1 and *E* ^*^ = *E*_*f*_ when *R*_0_ = 1. If *R*_0_ > 1, then we have the following theorem.

**Theorem 4 .**

If *R*_0_ > 1, then the chronic infection equilibrium *E* ^*^ is locally asymptotically stable.

**Proof**

We assume that *R*_0_ > 1. At *E* ^*^, (27) reduces to

(30) $$\begin{array}{c} \xi^{4}+a_{1}\xi^{3}+a_{2}\xi^{2}+a_{3}\xi +a_{4}=0, \end{array}$$

where

(31) a1=u+N+ϕ1,a2=Nu+ϕ1+ud+βv∗x∗+y∗+ϕ2+My∗p+q,a3=Nuϕ1+ϕ2+uϕ3+ϕ4+My∗up+q,a4=uNϕ3+ϕ4,

with

(32) M=cωz∗ω+y∗2,N=μ−cy∗ω+y∗,C1=βv∗y∗x∗+y∗2,C2=βv∗x∗x∗+y∗2+qz∗,ϕ1=a+d+C1+C2+pz∗,ϕ2=dC2+d+C1a+pz∗,ϕ3=C1a+pz∗+dβv∗x∗+y∗,ϕ4=My∗p+qd+pC1.

Clearly when *R*_0_ > 1, *a*_1_, *a*_2_, *a*_3_, and *a*_4_ are positive. In addition,

(33) a11a3a2=udC2+a+d+pz∗ +Nau+ad+a2+ϕ2+dϕ1 +dϕ2+a2a+C2+C1a2−pMy∗>0.

In the same manner, we have

(34) $$\begin{array}{c} \left|\begin{array}{ccc} a_{1} & 1 & 0\\ a_{3} & a_{2} & a_{1}\\ 0 & a_{4} & a_{3} \end{array}\right|=a_{3}\left|\begin{array}{cc} a_{1} & 1\\ a_{3} & a_{2} \end{array}\right|-a_{1}^{2}a_{4}>0. \end{array}$$

From the Routh-Hurwitz theorem given in [12], all roots of (30) have negative real parts. Then *E* ^*^ is locally asymptotically stable when *R*_0_ > 1.

## 4. Global Stability of Equilibria

In this section, we establish the global stability of the equilibria. Firstly, we have the following result.

**Theorem 5 .**

The disease-free equilibrium *E*_*f*_ is globally asymptotically stable when *R*_0_ ≤ 1.

**Proof**

Define

(35) $$\begin{array}{c} D=\left\{\left(x,y,v,z\right)\in R_{+}^{4}:z\geq \frac{b}{\mu }\right\}. \end{array}$$

We see that any solution (*x*(*t*), *y*(*t*), *v*(*t*), *z*(*t*)) starting in *D* remains there forever. Indeed, from Theorem 1 we get that (*x*(*t*), *y*(*t*), *v*(*t*), *z*(*t*)) ∈ *ℝ*_+_^4^. It remains to prove that *z*(*t*) ≥ *b*/*μ* with *z*_0_ ≥ *b*/*μ*. From the fourth equation of (3), we get

(36) $$\begin{array}{c} z\left(t\right)\geq \frac{b}{\mu }+\left(z_{0}-\frac{b}{\mu }\right)e^{-\mu t}. \end{array}$$

This implies that *z*(*t*) ≥ *b*/*μ*. Hence (*x*(*t*), *y*(*t*), *v*(*t*), *z*(*t*)) ∈ *D*.

If *R*_0_ ≤ 1, let us define a function *V* on *D* as follows:

(37) $$\begin{array}{c} V=y+\frac{\beta }{uR_{0}}v. \end{array}$$

Calculating the time derivative of *V* along the solution of (3), we obtain

(38) V˙=βR0xx+yR0−1v−p+qz−bμy,≤βR0R0−1v.

Since *R*_0_ ≤ 1, then $\dot{V}\leq 0$. Furthermore, if *S* is the set of solutions of the system, where $\dot{V}=0$, then the Lyapunov-LaSalle theorem [13] implies that all paths in *D* approach the largest positively invariant subset of the set *S*. Here, *S* is the set, where *v* = 0. On the boundary of *D*, where *v* = 0, we have *y* = 0, $\dot{x}=\lambda -dx$, and $\dot{z}=b-\mu z$. Then

(39) $$\begin{array}{c} \underset{t\to +\infty }{\lim}x\left(t\right)=\frac{\lambda }{d},\;\;\underset{t\to +\infty }{\lim}z\left(t\right)=\frac{b}{\mu }.\; \end{array}$$

Thus, all solution paths in *D* approach the disease-free equilibrium *E*_*f*_ when *R*_0_ ≤ 1. Hence, *E*_*f*_ is globally asymptotically stable in *D*.

To study the global stability of the chronic infection equilibrium, we will use the geometric approach defined by Li and Muldowney in [14]. A short overview of this geometric approach can be found in [15–17]. In a more simple way, Theorem  5.3 in [14] requires three conditions ensuring that global stability of a given equilibrium point is verified.

 
*The first condition* is the existence of a unique locally stable endemic equilibrium. Indeed, as proved in Theorem 4 from this paper, *E* ^*^ is the unique locally stable endemic equilibrium when *R*_0_ > 1.
 
*The second condition* is the existence of a compact set in the interior of the definition domain of the solutions *D* defined in the proof of Theorem 5, which is absorbing for the system (3). This is equivalent as shown in [18] to the uniform persistence of the state variables and the boundness of *D*. In our case, we proved in Theorem 1 that all solutions in system (3) are bounded. Thus the set *D* is also bounded. Further, we have proved in Theorem 3 that the disease-free equilibrium *E*_*f*_ is unstable if *R*_0_ > 1. This instability of *E*_*f*_ on ∂*D* implies the uniform persistence [19].
 
*The third condition* is the fulfillment of the Bendixson criterion [14]. In order to verify this third condition, we consider the following subsystem of (3):
  (40) x˙=λ−dx−βvxx+y+qyz,y˙=βvxx+y−ay−(p+q)yz,v˙=ky−uv.

The Jacobian matrix of system (40) is

(41) $$\begin{array}{c} J=\left(\begin{array}{ccc} -d-\frac{\beta vy}{{\left(x+y\right)}^{2}} & \frac{\beta vx}{{\left(x+y\right)}^{2}}+qz & -\frac{\beta x}{x+y}\\ \frac{\beta vy}{{\left(x+y\right)}^{2}} & -\frac{\beta vx}{{\left(x+y\right)}^{2}}-a-\left(p+q\right)z & \frac{\beta x}{x+y}\\ 0 & k & -u \end{array}\right) \end{array}$$

and its second addictive compound matrix is

(42) $$\begin{array}{c} J^{\left[2\right]}=\left(\begin{array}{ccc} -d-\frac{\beta v}{x+y}-a-\left(p+q\right)z & \frac{\beta x}{x+y} & \frac{\beta x}{x+y}\\ k & -d-\frac{\beta vy}{{\left(x+y\right)}^{2}}-u & \frac{\beta vx}{{\left(x+y\right)}^{2}}+qz\\ 0 & \frac{\beta vy}{{\left(x+y\right)}^{2}} & -\frac{\beta vx}{{\left(x+y\right)}^{2}}-a-\left(p+q\right)z-u \end{array}\right). \end{array}$$

In this case, we choose *P* = diag⁡(1, *y*/*v*, *y*/*v*). Hence,

(43) $$\begin{array}{c} P_{f}P^{-1}=\mathrm{diag}\left(0,\frac{\dot{y}}{y}-\frac{\dot{v}}{v},\frac{\dot{y}}{y}-\frac{\dot{v}}{v}\right), \end{array}$$

where matrix *P*_*f*_ is obtained by replacing each entry *p*_*ij*_ of *P* by its derivative in the direction of solution of (40). Moreover, we have

(44) $$\begin{array}{c} B=P_{f}P^{-1}+PJ^{\left[2\right]}P^{-1}=\left(\begin{array}{cc} B_{11} & B_{12}\\ B_{21} & B_{22} \end{array}\right), \end{array}$$

where

(45) B11=−d−βvx+y−a−(p+q)z,B12=βvxy(x+y)βvxy(x+y),B21=kyv0,B22=y˙y−v˙v−d−u−βvyx+y2βvxx+y2+qzβvyx+y2y˙y−v˙v−a−p+qz−u−βvxx+y2.

Let (*w*_1_, *w*_2_, *w*_3_) be a vector in *ℝ* ^3^; choose a norm in *ℝ* ^3^ defined as follows: |*w*_1_, *w*_2_, *w*_3_| = max⁡{|*w*_1_|, |*w*_2_| + |*w*_3_|} and let *μ* be the Lozinskii measure with respect to this norm. Then we have the following estimate; see [20]:

(46) $$\begin{array}{c} \mu \left(B\right)\leq \sup\left\{g_{1},g_{2}\right\}, \end{array}$$

where *g*_1_ = *μ*_1_(*B*_11_)+|*B*_12_| and *g*_2_ = |*B*_21_ | +*μ*_1_(*B*_22_); here *μ*_1_ denotes the Lozinskii measure with respect to *l*_1_ vector norm and |*B*_12_| and |*B*_21_| are matrix norms with respect to *l*_1_ norm. Moreover, we have

(47) μ1B11=−d−βvx+y−a−p+qz,B12=βvxyx+y=y˙y+a+p+qz,B21=kyv=v˙v+u,μ1B22=max⁡⁡y˙y−v˙v−d−u,y˙y−v˙v−a−u−p+qz≤y˙y−v˙v−u−δ.

Hence, we obtain

(48) g1=y˙y−d−βvx+y,g2≤y˙y−δ.

Therefore,

(49) $$\begin{array}{c} \mu \left(B\right)\leq \frac{\dot{y}}{y}-\delta . \end{array}$$

Consequently,

(50) $$\begin{array}{c} {\bar{q}}_{2}=\underset{t\to \infty }{\lim}\sup\underset{X_{0}\in K}{\sup}\frac{1}{t}\overset{t}{\underset{0}{\int }}\mu \left(B\right)ds\leq -\frac{\delta }{2}<0, \end{array}$$

which implies that the third condition is realized. Hence, the conditions of Theorem  3.5 in [14] are fulfilled; consequently, the endemic equilibrium (*x* ^*^, *y* ^*^, *v* ^*^) of the subsystem (40) is globally asymptotically stable.

Now, consider the fourth equation of system (3)

(51) $$\begin{array}{c} \dot{z}=b+\frac{cyz}{\omega +y}-\mu z, \end{array}$$

and its limit system is

(52) $$\begin{array}{c} \dot{z}=b+\frac{cy^{*}z}{\omega +y^{*}}-\mu z. \end{array}$$

Since *μ* − *cy* ^*^/(*ω* + *y* ^*^) = *b*/*z* ^*^, we get

(53) $$\begin{array}{c} \dot{z}=b\left(1-\frac{z}{z^{*}}\right). \end{array}$$

Therefore,

(54) $$\begin{array}{c} \underset{t\to \infty }{\lim}z\left(t\right)=z^{*}. \end{array}$$

Thus, the endemic equilibrium *E* ^*^ is globally asymptotically stable.

Summarizing the above, we have established the following result.

**Theorem 6 .**

The chronic infection equilibrium *E* ^*^ is globally asymptotically stable if *R*_0_ > 1.

## 5. Conclusion and Discussion

In this paper, we have presented a mathematical model based on a nonlinear system of differential equations. The population cells were partitioned into four classes, uninfected target cells, infected cells, free virus, and CTL cells. The basic reproduction number *R*_0_ corresponding to our model is independent of the liver size. Then, our model is more reasonable than the model presented in [2] to describe the HBV infection. In addition, we have proved the existence, positivity, and the boundedness of solutions of the problem, which implies that the model is well posed. By analyzing the model, we have shown that the disease-free equilibrium *E*_*f*_ is globally asymptotically stable if the basic reproduction number satisfies *R*_0_ ≤ 1, which leads to the eradication of virus from the liver. When *R*_0_ > 1, the disease-free equilibrium becomes unstable and a unique chronic infection equilibrium exists and is globally asymptotically stable. In this case, the virus persists in the population.

From our main results summarized above, we conclude that the dynamical behavior of our model is completely determined by the basic reproduction number *R*_0_. This allows determining the strategies to control the HBV infection by reducing the value of *R*_0_ to below or equal one (the case when *E*_*f*_ is globally asymptotically stable). From the explicit formula (4) for *R*_0_, we see that *R*_0_ can be decreased by increasing the export of precursor CTL cells from the thymus and both cytolytic and noncytolytic mechanisms. This observation shows that the CTL immune response plays a critical role in eradication of virus from the liver. On the other hand, *R*_0_ can be decreased by decreasing the parameters *β* and *k* which represent the rates of infection and production of virus, respectively. To do this biologically, we improve better our model by introducing the nucleoside analogues lamivudine or adefovir dipivoxil drug treatment in order to stop the virus from replicating. In addition, nucleoside analogues may also interfere with de novo infection of hepatocytes by hindering the transformation of relaxed circular DNA into cccDNA [21]. So, under therapy both production rate of new virions (*k*) and the rate of de novo infection (*β*) are reduced. Consequently, our model becomes

(55) x˙=λ−dx−1−ηβvxx+y+qyz,y˙=1−ηβvxx+y−ay−p+qyz,v˙=1−ϵky−uv,z˙=b+cyzω+y−μz,

where the parameters *η* and *ϵ* measure the efficacy of the therapy. An efficacy of 0 (0%) denotes that there is no inhibition, whereas an efficacy of 1 (100%) denotes complete inhibition. The basic reproduction number *R*_0*T*_ under therapy becomes

(56) $$\begin{array}{c} R_{0T}=\frac{\left(1-\epsilon \right)\left(1-\eta \right)\beta k}{u\left(a+\left(p+q\right)\left(b/\mu \right)\right)}, \end{array}$$

which implies that the basic reproduction number can be decreased by increasing the efficacy of drug treatment. Therefore, the results obtained from this work can be useful to determine an effective treatment against the hepatitis B virus.
